# Understanding the Role of Gibberellic Acid and Paclobutrazol in Terminal Heat Stress Tolerance in Wheat

**DOI:** 10.3389/fpls.2021.692252

**Published:** 2021-08-19

**Authors:** Shivani Nagar, V. P. Singh, Ajay Arora, Rajkumar Dhakar, Neera Singh, G. P. Singh, Shashi Meena, Sudhir Kumar, R. Shiv Ramakrishnan

**Affiliations:** ^1^Division of Plant Physiology, ICAR-Indian Agricultural Research Institute, New Delhi, India; ^2^Division of Agricultural Physics, ICAR-Indian Agricultural Research Institute, New Delhi, India; ^3^Division of Agricultural Chemistry, ICAR-Indian Agricultural Research Institute, New Delhi, India; ^4^ICAR-Indian Institute of Wheat and Barley Research, Karnal, India; ^5^College of Agriculture, Jawaharlal Nehru Krishi Vishwa Vidyalaya, Jabalpur, India

**Keywords:** gibberellic acid, wheat, terminal heat tolerance, reactive oxygen species, cell expansin

## Abstract

Understanding the physiological mechanism of tolerance under stress conditions is an imperative aspect of the crop improvement programme. The role of plant hormones is well-established in abiotic stress tolerance. However, the information on the role of gibberellic acid (GA) in abiotic stress tolerance in late sown wheat is still not thoroughly explored. Thus, we aimed to investigate the role of endogenous GA_3_ level in stress tolerance in contrasting wheat cultivars, *viz*., temperature-tolerant (HD 2643 and DBW 14) and susceptible (HD 2189 and HD 2833) cultivars under timely and late sown conditions. We created the variation in endogenous GA_3_ level by exogenous spray of GA_3_ and its biosynthesis inhibitor paclobutrazol (PBZ). Tolerant genotypes had higher antioxidant enzyme activity, membrane stability, and photosynthesis rate, lower lipid peroxidase activity, and better growth and yield traits under late sown conditions attributed to H_2_O_2_ content. Application of PBZ escalated antioxidant enzymes activity and photosynthesis rate, and reduced the lipid peroxidation and ion leakage in stress, leading to improved thermotolerance. GA_3_ had a non-significant effect on antioxidant enzyme activity, lipid peroxidation, and membrane stability. However, GA_3_ application increased the test weight in HD 2643 and HD 2833 under timely and late sown conditions. GA_3_ upregulated GA biosynthesis and degradation pathway genes, and PBZ downregulated kaurene oxidase and GA_2_ox gene expression. GA_3_ also upregulated the expression of the cell expansins gene under both timely and late sown conditions. Exogenous GA_3_ did not increase thermotolerance but positively affected test weight and cell expansins gene expression. No direct relationship existed between endogenous GA_3_ content and stress tolerance traits, indicating that PBZ could have conferred thermotolerance through an alternative mechanism instead of inhibiting GA_3_biosynthesis.

## Introduction

Sustainable food production is always at increased risk due to abiotic stresses. The high-temperature stress, especially at the reproductive stage, i.e., terminal heat stress, poses a threat to global crop production. Zhao et al. (2017) estimated that a unit-degree Celsius increase in global mean temperature would, on average, reduce global yields of wheat by 6.0%, rice by 3.2%, maize by 7.4%, and soybean by 3.1%. Wheat is the second-largest source of global human calorie intake. Terminal heat stress in wheat propounds a challenge to the scientific community in the present and future climate change scenarios. The abnormal delay in sowing wheat in the Indian rice-wheat belt exposes it to unsuited photothermal regimes at all phenophases. A crop simulation model-based study by Dubey et al. (2020) predicted terminal heat stress would reduce Indian wheat yield by 18.1, 16.1, and 11.1% in the present, 2020, and 2050 scenarios, respectively. Terminal heat stress at the time of anthesis and grain filling stage causes flower abortion, reduced pollen viability, availability and translocation of photosynthates to the developing kernel, and starch synthesis and its deposition within the kernel, thus resulting in lower grain number, grain weight, and grain quality (Farooq et al., 2011; Reynolds et al., 2012; Nagar et al., 2015).

High-temperature stress increases the availability of free electrons in chloroplast and mitochondria, leading to the generation of Reactive oxygen species (ROS) (O${\;}_{2}^{-}$, H_2_O_2_, OH^−^, • OH) (Foyer et al., 1997; Caverzan et al., 2016). These ROS cause damage to lipids, nucleic acid, and protein, leading to a decrease in the overall efficiency of the physiological processes of the plant (Mathur and Jajoo, 2014). When under stress, the degradation of chlorophyll pigments causes a significant reduction in photosynthesis and reversible effects on decrease in CO_2_ solubility, enzymatic activities of rubisco and rubisco activase, and structural damage to thylakoid membranes and oxygen-evolving complex of photosystem II (Blum and Ebercon, 1981; Harding et al., 1990; Farooq et al., 2011).

In response to high-temperature stress, plants have evolved a highly responsive mechanism of temperature sensing and signaling. An increase in temperature is perceived by membrane-bound receptors directly and in terms of change in membrane fluidity as membrane lipids reach their melting points. Calcium channels become active and initiate the influx of Ca^2+^ ion. An increase in concentration of Ca^2+^ ion, misfolded/unfolded protein, ROS, and stress-induced histone modification causes activation of signal transduction and transcription factor. In response to high-temperature stress, plants activate stress-adaptive physiological responses, i.e., increase in the level of stress proteins (heat shock transcription factors, heat shock protein, and drought response element-binding protein) and production of antioxidant enzymes and ROS scavengers. Further, the change in the fatty acid composition of membrane lipids, accumulation of compatible solutes and increase in levels of stress protecting hormone also provides stress tolerance (Wahid et al., 2007; Sajid et al., 2018).

Plant hormones play a pivotal role in the regulation of plant growth and development. However, their role becomes more crucial under stress because they also act as stress signaling molecules, response determiners, and regulators. Thus, phytohormones are considered the most critical endogenous substances because they regulate many physiological and developmental processes. They also have a role in developing stress tolerance by affecting/regulating them directly and indirectly. The role of phytohormones like abscisic acid, cytokinins, auxins, and salicylic acid is well-established under abiotic stress (Wahid et al., 2007). Gibberellic acid (GA) is a crucial plant hormone that is essential for plants throughout their life cycle. It regulates process such as seed germination, leaf expansion, stem elongation, flower and trichome initiation, source–sink relationship and flower, fruit and seed development (Yamaguchi, 2008; Iqbal et al., 2011). Many workers have reported reduced GA levels and signaling under stress (Ahmad, 2010; Yang et al., 2013; Abdulaziz et al., 2020).

We encountered enough evidence of exogenous application of GA_3_ on various plant growth and physiological processes under salinity (Maggio et al., 2010; Forghani et al., 2020), drought (Kaya et al., 2006; Moumita Al Mahmud et al., 2019), and cold and osmotic stresses (Skirycz et al., 2011; Claeys et al., 2012). GA modulated the oxidative stress processes and antioxidant enzyme activity, consequently suppressing the negative effect of abiotic stress (Khan et al., 2012). In addition to the antioxidant defense system, exogenous GA upregulated the glyoxalase system, which assisted the survival of wheat seedlings under drought stress (Moumita Al Mahmud et al., 2019). Unexpectedly, few reports are available to witness the vital roles of GAs in high-temperature stress response and adaptation, especially at the reproductive stage. Paclobutrazol (PBZ), a triazole derivative, inhibits GA biosynthesis in the plant by inhibiting kaurene oxidase and blocking oxidation of kaurene to kaurenoic acid. PBZ can increase cold, heat, drought, and salt resistance in various plants. The triazole-mediated stress protection is due to changes in hormones such as an increase in cytokinins, a transient rise in ABA, and a decrease in ethylene (Soumya et al., 2017). It helps in plant stress adoption by maintaining relative water content and electrolyte leakage, and protecting the photosynthetic machinery by enhancing the levels of osmolytes, antioxidant activities, and endogenous hormones; thereby enhancing photosynthetic activity contributing to plant growth and yield (Pinhero and Fletcher, 1994; Desta and Amare, 2021). Here, we present a comparative study to understand the mechanism of PBZ- and GA_3_-mediated stress tolerance in wheat. At the reproductive stage, contrasting spring wheat cultivars, i.e., heat stress-sensitive and tolerant plants, were exposed to terminal heat stress by changing the sowing date. We applied GA_3_ and PBZ through foliar application in the same experiment set to create the variation in endogenous GA level. We studied that, whether the level of endogenous GA regulated the trait determining the antioxidant system, photosynthesis, stress tolerance ability and finally yield attributes in late sown wheat. The knowledge gained through this study would provide a better insight into the role and mechanism of GA_3_-mediated heat stress tolerance ability at a reproductive stage, which may play a crucial role in crop improvement for abiotic stress tolerance through breeding and biotechnological approaches.

## Materials and Methods

### Plant Growth Condition and Stress Treatment

Four wheat genotypes—*viz*., heat-tolerant HD 2643 and DBW 14, and heat-susceptible HD 2189 and HD 2833—were selected based on initial screening of 40 wheat genotypes for heat stress tolerance and susceptibility under late sown condition (Nagar et al., 2015). Genotypes were grown under open field conditions at the Division of Plant Physiology, Indian Agricultural Research Institute, New Delhi, India during Nov-April, 2014–15. The sowing was done on 18th Nov (Timely Sown; TS) and 5th Jan (Late Sown; LS) to expose plants to different temperature regimes (Supplementary Table 1). Pots were filled with 15-kg mixture of clay loam soil and farmyard manure (3:1). Farmyard manure (decomposed mixture of dung and urine of farm animals) is a good source of organic carbon, and it also contains 0.5% N, 0.2% P_2_O_5_, and 0.5% K_2_O. Fertilizers, i.e., urea, single super phosphate, and muriate of potash were applied, respectively, at sowing (N:P:K in the dose of 60:60:60 kg ha^−*l*^). The remaining 60 kg, N ha^−1^ was applied in the form of urea at 25 days after sowing. During the whole crop growing season, plants were maintained under well-watered and pest-free conditions. On average, LS wheat faced 3.82°C higher temperature during booting to anthesis and 5.96°C higher temperature from 50% anthesis to maturity compared with TS plants (Supplementary Table 2). At 5 days after booting and 50% anthesis, the PBZ (50 ppm) and gibberellic acid (100 ppm)—concentrations were finalized based on previous studies and preliminary work in our lab—applied. The mock solution was sprayed on the control plant. At 10 days after anthesis, the physiological study and enzyme activity in flag leaf was estimated. Plants were tagged at the time of booting and 50% anthesis to maintain uniformity in spray and sampling plants.

GA_3_ extraction and gene expression studies in developing grains of the wheat spike were conducted. Main spikes were identified and tagged on 50% anthesis.

### Growth and Yield

At the time of crop maturity, data related to total dry weight, plant height, number of tillers per plant, grain weight per ear, grain number per ear, test weight, and grain weight per plant were recorded. Test weight is a good indicator of grain quality and was measured as the average weight of 1,000 seeds. Harvest index was also calculated, and the ratio of the economic yield to biological yield was expressed as a percentage.

### Membrane Stability Index (MSI)

MSI was measured as a function of electrolyte leakage by Sairam et al. (1997). Initial conductivity (C_1_) at 40°C and final conductivity (C_2_) at 100°C from leaf leachate were recorded on a conductivity bridge. MSI was calculated as: MSI = [1 - (C_1_/C_2_)] × 100.

### H_2_O_2_ Content

Leaf samples were homogenized with Liq. N_2_, followed by the addition of 5 ml cooled acetone. The mixture was centrifuged at 12,000 rpm, 4°C for 20 min and the supernatant was collected. In supernatant, 2 ml titanium reagent (Teranishi et al., 1974) and 2.5 ml liquid ammonia solution were added to precipitate the titanium-hydro peroxide complex. The reaction mixture was centrifuged at 10,000 g for 10 min at 4°C. The precipitate was dissolved in 5 ml of 2 M H_2_SO_4_ and read at 415 nm in UV-visible spectrophotometer against reagent blank, and the concentration of H_2_O_2_ was expressed as μmol H_2_O_2_g^−1^ DW.

### Lipid Peroxidation

Standard protocol of Heath and Packer (1968) was used, which measured the formation of thiobarbituric acid reactive substances. Leaf sample was homogenized in 0.1% TCA and centrifuged at 10,000 g for 15 min. Take one ml supernatant and add 4.0 mL of 0.5% thiobarbituric acid prepared in 20% Trichloroacetic acid. The mixture was heated at 95°C for 30 min followed by cooling in an ice bath. After centrifugation at 10,000 g for 10 min, the specific and non-specific absorbance of the supernatant was recorded at 532 and 600 nm, respectively. The concentration of malonaldehyde was calculated using an extinction coefficient of 155 mM^−1^ cm^−1^.

### Antioxidant Enzymes Estimation

Extraction of enzymes superoxide dismutase (SOD), catalase, and glutathione reductase (GR) was done by grinding leaf samples in liquid N_2_, followed by adding extraction buffer consisting of 100 mM phosphate buffer, which contained 0.5 mM EDTA, pH 7.5. For ascorbate peroxidase (APX), the ascorbic acid was added to the extraction buffer in the final concentration of 1 mM, and pH was further adjusted to 7.5. Leaf extracts were centrifuged at 12,000 rpm at 4°C for 20 min, and the supernatant was collected for enzyme estimation. SOD activity was estimated in terms of decrease in absorbance of formazone at 540 nm made by nitro-blue tetrazolium with O^2−^ radical due to SOD (Dhindsa et al., 1981). The method by Aebi (1984) was used to measure catalase activity. Breakdown of H_2_O_2_ by catalase enzyme was recorded in terms of decreased absorbance at 240 nm after adding H_2_O_2_. The enzyme activity (μmol H_2_O_2_ reduced min^−1^ mg^−1^ protein) was calculated using 36.5 M^−1^cm^−1^ as the extinction coefficient of H_2_O_2_. GR activity was assayed as per method by Smith et al. (1988). The formation of reduced glutathione from oxidized glutathione in the presence of Nicotinamide adenine dinucleotide phosphate (NADH) was assayed in terms of the colored complex formed by reduced glutathione 5,5-dithiobis-2-nitrobenzoic acid (DTNB). An increase in absorbance at 412 nm was recorded, and enzyme activity (μmol oxidized glutathione formed min^−1^ mg^−1^ protein) was calculated using 6.2 mM^−1^cm^−1^ as extinction coefficient of oxidized glutathione. Ascorbate peroxidase (APOX) activity was assayed by method given by Nakano and Asada (1981). Ascorbic acid reduced hydrogen peroxide to water in the presence of 0.1 M phosphate buffer. A decrease in ascorbic acid was recorded at 240 nm. The enzyme activity (μmol H_2_O_2_ reduced min^−1^ mg^−1^ protein) was calculated using 2.8 mM^−1^cm^−1^ as extinction coefficient of H_2_O_2._

### Photosynthesis Rate

The portable Infrared Gas Analyzer (IRGA), LI-6400XT Model (Li-COR Ltd., Lincoln, Nebraska, USA) under standard operating condition (temperature: 30–35°C, relative humidity: 50–60%, CO_2_ concentration: 350 ± 50 μmol mol^−1^, and photosynthetic photon flux density: 1,200 μmol m^−2^s^−1^) was used to measure photosynthetic rate, Fv/Fm ratio, transpiration rate, and stomatal conductance on fully expanded flag leaf of each plant. These measurements were made between 10:15 and 11:45 a.m. at 10 days after athesis (DAA).

### Gibberellic Acid Extraction

Developing grains were ground into a fine powder and placed into screw cap tubes filled with 30 ml methanol 70% (v/v) and kept overnight at 4°C. The extract was centrifuged, and methanol was evaporated under a vacuum from the supernatant. The pH of the aqueous phase was adjusted to 8.5 and then partitioned with ethyl acetate. After removal of the ethyl acetate phase, the pH of the aqueous phase was adjusted to 2.5. The solution was partitioned with diethyl ether, and then passed through sodium sulfate. Diethyl ether was evaporated under vacuum, and dry residue containing GA_3_ was dissolved in 2.0 ml of absolute methanol. The GA_3_ analysis was performed using high performance liquid chromatography (HPLC) (Waters) equipped with reversed-phase column Crestpak C18 (150 × 4.6 mm i.d.; 5 μm) maintained at 30 ± 1°C. The mobile phase of acetonitrile-water (30:70%; v/v) was used with pH-4.5 and a flow rate of 1 ml/min. An injection volume of 10 μl was used for each analysis, and the wavelength used for analysis was 208 nm.

### Analysis of Gene Expression by RT-PCR and Real-Time PCR

Total RNA was extracted from developing seed (cell expansin gene) and leaf tissue (GA_3_ biosynthesis gene) using RNA easy kit (Qiagen Inc., Chatsworth, CA USA) according to the instructions of the manufacturer. DNase treatment was done to remove the DNA contamination (Ambion TURBO DNase, Thermo Fisher Scientific Waltham, MA, USA). RNA was reverse transcribed in a 20 μl reaction using cDNA Reverse Transcription Kit [SuperScript® III First-Strand Synthesis System (Invitrogen™, Carlsbad, CA, USA) according to the manual provided by the manufacturer. The details of NCBI gene accession number for specific gene and primers used in this study are given as Supplementary Table 3. For RT-PCR expression of GA biosynthesis pathway genes [ent-copalyl diphosphate synthase (CPS), ent-kaurene synthase, (EKS), ent-kaurene oxidase (KO), ent-kaurenoic acid oxidase (KAO), 20-oxoglutarate-dependent dioxygenase (GA20ox), 3 oxidase (GA3ox), 2 oxidase (GA2ox)], complementary DNA (cDNA) was normalized by using wheat actin as a control. Normalized quantity of cDNA was used for amplification (27–28 cycles standardized for genes). Specific genes were amplified using Invitrogen two-step RT-PCR Kit (Invitrogen™, Carlsbad, CA, USA) with gene-specific and degenerate forward and reverse primers. A real-time expression study was conducted by real-time quantitative PCR (RT-qPCR) with Power SYBR® Green Master Mix (Thermo fisher scientific, USA) in Stratagene Mx3005P (Agilent Technologies, USA). The relative expression levels in data were determined based on the 2–ΔΔCT method (Livak and Schmittgen, 2001) using wheat actin as an internal control.

### Statistical Analysis

The results for physiological, biochemical, and growth and yield traits in cultivars with varied heat-tolerance and GA-sensitivity were expressed as means with standard error (SE). All the statistical data analyses were performed using three biological replicates of physiological and biochemical traits for each treatment, while five biological replicates were taken for growth and yield traits. The three factorial Analysis of variance (ANOVA) for completely randomized design (CRD) consisting of genotype, stress, and spray as fixed factors was performed using the “aov” function available in the “stats” package of statistical software “R” version 4.04. The *P*-value of main and interaction effects for all the response variables is provided in Supplementary Table 4. LSD method (using LSD.test function in “agricolae” package of R) was used to analyse the differences between the means of the levels of a factor at *P* < 0.05.

## Results

### Endogenous GA3 Content

**Figure 1:**
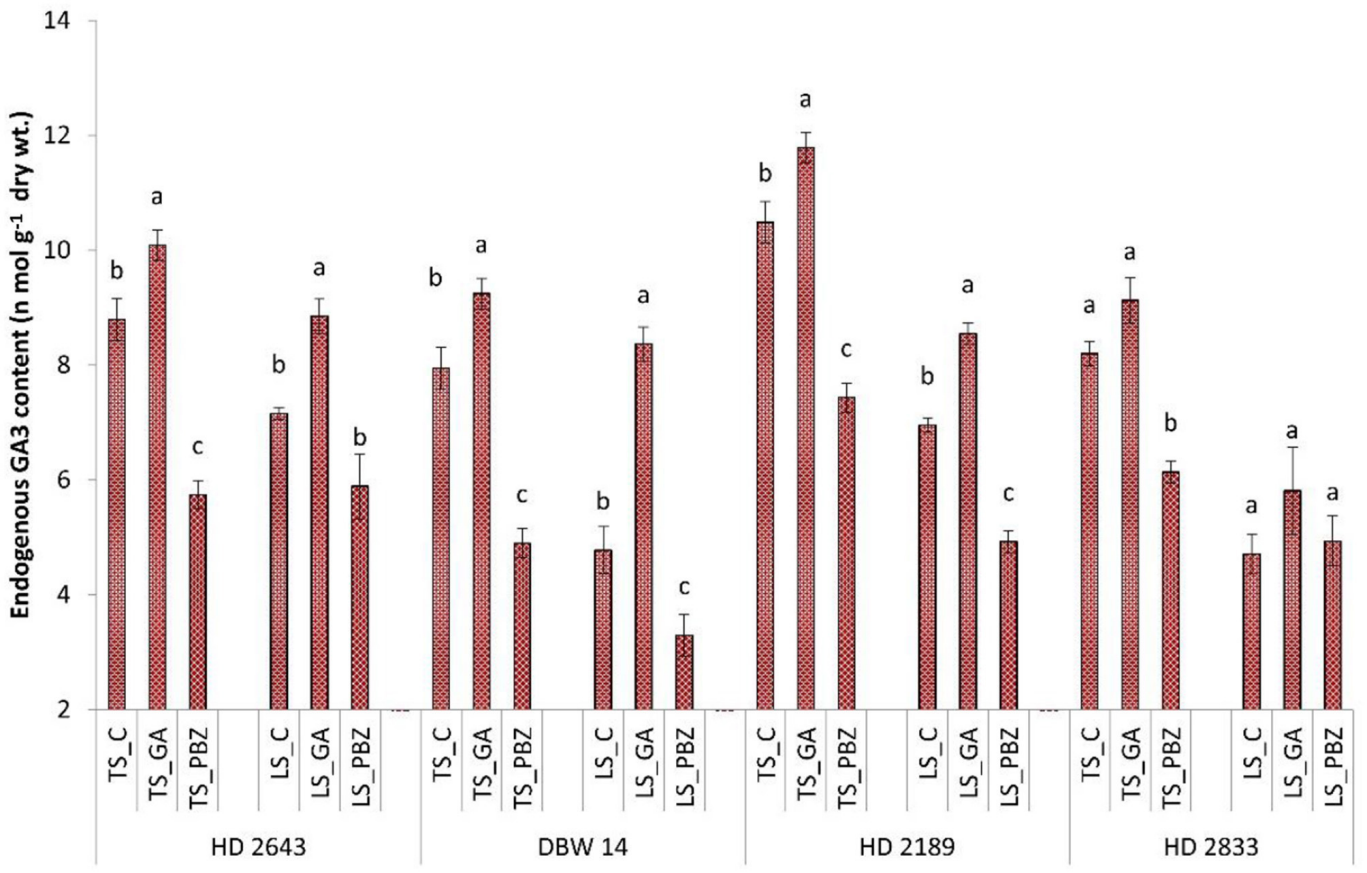
Effect of post-anthesis application of gibberellic acid (100 ppm) and paclobutrazol (50 ppm) on endogenous GA_3_ content (n mol g^−1^ dry wt) in wheat exposed to timely and late sown conditions. The different letters on bar denotes significant difference among the treatments within the given variety and stress level according to LSD test at *P* < 0.05. The error bar represents the standard error. Value on the ordinate is the mean of five replicates. On the abscissa, HD 2643, DBW 14, HD2189, and HD 2833 are varieties; TS and LS denote timely sown and late sown of stress treatment, respectively; C, GA, and PBZ denote control, GA3, and paclobutrazol of spray treatment, respectively.

It was observed that, in LS plants, average GA_3_ content decreased by 33% compared to TS plants (Figure 1). GA_3_ content was highest in HD 2643 and HD 2189 in stressed and non-stressed conditions, respectively. PBZ significantly (*P* < 0.05) decreased GA_3_ content by 32 and 18% in all cultivars under TS and LS conditions, respectively. Exogenous application of GA_3_ significantly (*P* < 0.05) increased GA_3_ content by 13.7 and 36% in all cultivars under TS and LS conditions, respectively.

### Growth and Yield Traits

In LS plants, the main effect was a significant decrease (*P* < 0.0001) in total dry weight, plant height, number of tillers per plant, grain weight and number per ear, test weight, grain weight per plant, and harvest index in all cultivars (Table 1, Supplementary Table 4). The ANOVA showed that contrasting varieties as a main effect is significant at *P* < 0.001 except ear per panicle and tiller per plant (Supplementary Table 4). Under the LS condition, tolerant cultivars had an average decrease of 21, 12, 12, 13, and 15% in total dry weight, grain weight per ear, grain number per ear, grain weight per plant, and harvest index; whereas, in susceptible cultivars, the decrease was by 26.6, 16, 17, 23, 25%, respectively. These results were statistically different at *P* < 0.05, where the average values of grain weight per ear ranged from 2.3 to 3.2 g for different cultivars under TS conditions, while it varied from 1.58 to 2.37 g under stressed conditions.

**Table 1:** Effect of post-anthesis application of gibberellic acid (100 ppm) and paclobutrazol (50 ppm) on plant height (cm), tiller number per plant, grain number/ear, grain weight per ear (g), test weight, grain yield (g/plant), and total dry weight (g/plant) in wheat exposed to timely and late sown conditions.

**Variety**	**Stress** ** _Spray**	**Plant height** **(cm)**	**Tiller number**	**Grain number/** **ear**	**Grain weight/** **ear (g)**	**Test weight** **(g)**	**Grain yield** **(g)**	**Total dry** **matter (g)**	**Harvest** **index (%)**
HD 2643	TS_C	118.33 ± 3.48^a^	7.33 ± 0.33^a^	62.33 ± 0.33^a^	2.79 ± 0.07^b^	42.33 ± 0.7^b^	18.05 ± 0.47^b^	40.33 ± 0.88^a^	55.86 ± 1.06^a^
	TS_GA	118.33 ± 0.88^a^	6.67 ± 0.33^a^	64 ± 0.58^a^	2.82 ± 0.06^b^	45.1 ± 0.4^a^	18.79 ± 0.42^b^	41.33 ± 2.03^a^	56.17 ± 0.67^a^
	TS_PBZ	116 ± 3.79^a^	7.33 ± 0.33^a^	63.67 ± 0.88^a^	3.25 ± 0.1^a^	45.99 ± 0.46^a^	20.92 ± 0.42^a^	40.07 ± 0.52^a^	56.56 ± 0.6^a^
	LS_C	105 ± 2.08^a^	7 ± 0.58^a^	54.33 ± 0.88^b^	2.04 ± 0.04^b^	37.02 ± 0.32^b^	15.1 ± 0.45^c^	32 ± 0.84^a^	45.98 ± 1.51^a^
	LS_GA	106 ± 4.16^a^	6.67 ± 0.33^a^	56.7 ± 0.88^a^	2.33 ± 0.06^a^	39.8 ± 0.38^a^	18.23 ± 0.55^b^	33.5 ± 0.81^a^	46.82 ± 0.79^a^
	LS_PBZ	105 ± 3.06^a^	7.33 ± 0.33^a^	57.9 ± 0.58^ab^	2.43 ± 0.05^a^	41.6 ± 0.35^a^	19.33 ± 0.58^a^	35.53 ± 1.34^a^	47.51 ± 1.5^a^
DBW 14	TS_C	97 ± 4.04^a^	8.67 ± 0.33^a^	57 ± 0.58^b^	3.21 ± 0.06^b^	40.61 ± 0.64^c^	20.74 ± 0.35^b^	38.58 ± 0.63^a^	56.74 ± 0.56^a^
	TS_GA	103.33 ± 4.26^a^	8.33 ± 0.88^a^	62 ± 0.58^a^	3.45 ± 0.08^a^	42.84 ± 0.2^b^	23.6 ± 0.7^a^	37.83 ± 0.35^a^	57.31 ± 0.86^a^
	TS_PBZ	101.33 ± 6.94^a^	8.67 ± 0.88^a^	57.67 ± 0.33^b^	3.52 ± 0.05^a^	45.16 ± 0.62^a^	24.37 ± 0.34^ab^	39.43 ± 0.69^a^	58.04 ± 0.92^a^
	LS_C	89 ± 2.65^a^	7.67 ± 0.33^a^	50.33 ± 0.88^b^	2.17 ± 0.13^b^	38.55 ± 0.74^b^	18.13 ± 0.48^b^	30.24 ± 0.76^a^	48.97 ± 1.75^a^
	LS_GA	88.33 ± 2.33^a^	7 ± 1^a^	52 ± 0.58^ab^	2.25 ± 0.1^ab^	38.4 ± 0.48^b^	19.29 ± 1.05^b^	30.45 ± 0.99^a^	51.72 ± 1.42^a^
	LS_PBZ	86.33 ± 1.45^a^	7 ± 0.58^a^	53.33 ± 0.88^a^	2.61 ± 0.09^a^	41.72 ± 0.54^a^	22.5 ± 0.7^a^	32.48 ± 1.14^a^	51.57 ± 1.65^a^
HD 2189	TS_C	86.33 ± 5.04^a^	7.33 ± 0.33^a^	61 ± 0.58^ab^	2.48 ± 0.02^b^	41.37 ± 0.49^b^	19.37 ± 0.27^c^	32.22 ± 1.26^a^	46.22 ± 1.22^a^
	TS_GA	88 ± 4.93^a^	7.33 ± 0.88^a^	62.33 ± 0.58^a^	2.56 ± 0.07^ab^	42.96 ± 0.65^ab^	21.57 ± 0.62^b^	31.96 ± 1.09^a^	47.04 ± 0.99^a^
	TS_PBZ	86.33 ± 1.45^a^	6.33 ± 0.33^a^	58.67 ± 1.2^b^	2.72 ± 0.07^a^	43.4 ± 0.4^a^	23.93 ± 0.17^a^	32.9 ± 1.92^a^	46.49 ± 2.38^a^
	LS_C	75.5 ± 0.87^a^	6.33 ± 0.33^a^	50.8 ± 0.88^a^	2.04 ± 0.05^c^	34.38 ± 0.57^c^	15.03 ± 0.46^b^	23.9 ± 0.82^a^	33.83 ± 2.69^a^
	LS_GA	76.33 ± 0.88^a^	6.33 ± 0.67^a^	54.33 ± 0.88^a^	2.51 ± 0.05^b^	37.89 ± 0.06^b^	17.99 ± 0.69^a^	22.15 ± 1.21^a^	34.32 ± 1.94^a^
	LS_PBZ	73 ± 1.53^a^	7 ± 0.58^a^	55.7 ± 0.88^a^	2.7 ± 0.05^a^	39.07 ± 0.63^a^	18.4 ± 0.1^a^	25.66 ± 1.27^a^	35.43 ± 1.27^a^
HD 2833	TS_C	78.33 ± 2.4^a^	7.67 ± 0.33^a^	59.67 ± 0.33^a^	2.34 ± 0.04^b^	38.71 ± 0.52^c^	16.03 ± 0.43^b^	31.8 ± 0.98^a^	50.66 ± 0.29^b^
	TS_GA	80.67 ± 2.33^a^	7.67 ± 0.67^a^	61.33 ± 0.58^a^	2.52 ± 0.05^a^	41.76 ± 0.77^b^	17.99 ± 0.16^a^	33.35 ± 0.78^a^	56.29 ± 1.07^a^
	TS_PBZ	78.33 ± 4.37^a^	7.33 ± 0.33^a^	60 ± 0.58^a^	2.66 ± 0.04^a^	44.46 ± 0.83^a^	18.4 ± 0.31^a^	33.49 ± 1.69^a^	55.51 ± 0.67^a^
	LS_C	67 ± 1.53^a^	6.67 ± 0.33^a^	49.33 ± 0.67^b^	1.58 ± 0.06^b^	34.02 ± 0.97^b^	12.23 ± 0.42^c^	23.1 ± 1.22^a^	39.4 ± 1.7^a^
	LS_GA	67.33 ± 2.33^a^	6.67 ± 0.33^a^	54 ± 0.58^a^	1.92 ± 0.02^a^	37.8 ± 1.12^a^	13.88 ± 0.56^b^	22.16 ± 0.87^a^	40.35 ± 2.55^a^
	LS_PBZ	67 ± 1.73^a^	6.33 ± 0.67^a^	51.33 ± 1.45^ab^	1.88 ± 0.08^a^	38.17 ± 0.35^a^	16.17 ± 0.37^a^	24.12 ± 1.05^a^	40.38 ± 0.93^a^

*The different letters (as superscript) in a column denotes significant difference among the treatments within the given variety and stress level according to LSD test at P < 0.05. The value follows ± represent the standard error (n = 3).*

*TS, Timely sown; LS, Late Sown; C, Control; GA, GA3 spray; PBZ, Paclobutrazol spray*.

An exogenous spray of GA_3_ and PBZ had a statistically non-significant (*P* < 0.05) effect on total biomass, the number of tillers per plant, and the height of the plant in all cultivars under both sowing conditions. However, the harvest index increased significantly only in TS HD 2833. Application of GA_3_ and PBZ showed varietal variation for grain number per ear under TS condition. In stress plants, however, improvement in grain number per ear was recorded with the application of growth regulators. The application of GA_3_ enhanced grain weight per ear, grain number per ear, test weight, and grain yield per plant by 13.4, 3.8, 3.6, and 13.6% in the tolerant cultivars, and by 22.2, 8.2, 10, and 16.5% in susceptible cultivars under stress, respectively. Application of GA_3_ and PBZ significantly (*P* < 0.05) increased test weight and grain yield per plant in all cultivars under LS condition, except in a few cases [DBW 14 showed non-significant (NS) effect of GA_3_ in the case of test weight and PBZ on grain yield]. In LS plants, application of PBZ enhanced grain weight per ear, grain number per ear, test weight, and grain yield per plant by 21, 6.3, 10.3, and 26.5% in the tolerant cultivars, and by 25.6, 6.8, 12.9, and 16.9% in the susceptible cultivars under stress, respectively.

### Photosynthesis Rate and Related Traits

The photosynthesis rate and associated traits showed statistically significant difference (*P* < 0.001) in variety, stress, and spray as a main effect (Supplementary Table 4). Under stress, a decrease of 42 and 45% was recorded in mean photosynthesis (values averaged over varieties of contrasting group) in tolerant and susceptible cultivars, respectively. Photosynthesis rate was lowest in HD 2189 and highest in DBW 14 under both environmental conditions. PBZ augmented photosynthesis rate by 6% (NS at *P* < 0.05) and 22% (significant at *P* < 0.05) in TS and LS plants of tolerant cultivars and by 9% (significant at *P* < 0.05) and 13% (significant at *P* < 0.05) in susceptible cultivars, respectively. An increase of 5.1/6.6% (NS at *P* < 0.05) in photosynthesis rate on GA_3_ application was observed in TS/LS tolerant cultivars. In comparison, there was a 2.6% (NS at *P* < 0.05)/13% (significant at *P* < 0.05) increase in susceptible cultivars (Table 2). Stress conditions reduced photochemical efficiency by 20% (significant at *P* < 0.05), with a more considerable reduction in susceptible cultivars (26.4% significant at *P* < 0.05) than in tolerant cultivars (13.7% significant at *P* < 0.05). PBZ application led to a significant increase in photochemical efficiency under most of the treatments. On the contrary, GA_3_ application significantly (*P* < 0.05) increased photochemical efficiency under few treatments such as TS HD 2643 and HD 2189 and LS HD 2189.

**Table 2:** Effect of post-anthesis application of gibberellic acid (100 ppm) and paclobutrazol (50 ppm) on photosynthesis rate (μmoles CO_2_ m^−2^ s^−1^), Fv/Fm ratio, transpiration rate (mmol cm^−2^ s^−1^), and stomatal Conductance (cm s^−1^) in wheat exposed to timely and late sown conditions.

**Variety**	**Stress_Spray**	**Photosynthesis rate** **(μmoles CO2 m** ^**–2**^ **s** ^**–1**^ **)**	**Fv/Fm ratio**	**Transpiration rate** **(mmol cm**^**−2**^ s^**–1**^**)**	**Stomatal conductance** **(cm s** ^**–1**^ **)**
HD 2643	TS_C	25.33 ± 0.67^a^	0.76 ± 0.001^c^	0.24 ± 0.003^b^	3.49 ± 0.019^b^
	TS_GA	26.67 ± 0.33^a^	0.79 ± 0.003^b^	0.25 ± 0.003^ab^	3.51 ± 0.035^b^
	TS_PBZ	27 ± 0.58^a^	0.8 ± 0^a^	0.26 ± 0.007^a^	3.97 ± 0.072^a^
	LS_C	14.67 ± 0.33^b^	0.67 ± 0.004^a^	0.32 ± 0.006^b^	4.15 ± 0.026^b^
	LS_GA	15 ± 0.58^b^	0.68 ± 0.007^a^	0.32 ± 0.003^b^	4.47 ± 0.083^a^
	LS_PBZ	17.33 ± 0.33^a^	0.68 ± 0.003^a^	0.34 ± 0.009^a^	4.59 ± 0.013^a^
DBW 14	TS_C	26 ± 0.58^b^	0.78 ± 0.004^b^	0.33 ± 0.006^b^	3.53 ± 0.047^b^
	TS_GA	27.33 ± 0.33^ab^	0.78 ± 0.004^ab^	0.35 ± 0.003^ab^	3.55 ± 0.034^b^
	TS_PBZ	27.67 ± 0.33^a^	0.79 ± 0.002^a^	0.35 ± 0.006^a^	3.74 ± 0.046^a^
	LS_C	15 ± 0.58^b^	0.66 ± 0.004^b^	0.44 ± 0.003^a^	4.29 ± 0.053^a^
	LS_GA	16.67 ± 0.88^ab^	0.67 ± 0.001^b^	0.44 ± 0.006^a^	4.39 ± 0.043^a^
	LS_PBZ	19 ± 0.58^a^	0.69 ± 0.005^a^	0.47 ± 0.012^a^	4.47 ± 0.06^a^
HD 2189	TS_C	23 ± 0.58^b^	0.78 ± 0.004^c^	0.24 ± 0.007^b^	3.4 ± 0.037^b^
	TS_GA	22.67 ± 0.33^b^	0.81 ± 0.005^b^	0.24 ± 0.006^b^	3.47 ± 0.026^b^
	TS_PBZ	25.67 ± 0.33^a^	0.85 ± 0.003^a^	0.27 ± 0.003^a^	3.64 ± 0.067^a^
	LS_C	12.67 ± 0.33^a^	0.57 ± 0.006^c^	0.33 ± 0.003^a^	4.46 ± 0.052^a^
	LS_GA	14.33 ± 0.88^a^	0.61 ± 0^b^	0.34 ± 0.003^a^	4.62 ± 0.041^a^
	LS_PBZ	13.67 ± 0.88^a^	0.62 ± 0.006^a^	0.34 ± 0.006^a^	4.52 ± 0.058^a^
HD 2833	TS_C	25 ± 0.58^b^	0.8 ± 0.006^a^	0.3 ± 0.003^b^	2.71 ± 0.042^b^
	TS_GA	26.67 ± 0.33^a^	0.8 ± 0.001^a^	0.31 ± 0.006^ab^	2.88 ± 0.038^ab^
	TS_PBZ	26.67 ± 0.33^a^	0.81 ± 0.004^a^	0.32 ± 0.006^a^	3 ± 0.094^a^
	LS_C	14 ± 0.58^b^	0.59 ± 0.005^b^	0.33 ± 0.003^b^	3.33 ± 0.034^b^
	LS_GA	16 ± 0.58^a^	0.6 ± 0.006^ab^	0.33 ± 0.003^b^	3.35 ± 0.026^b^
	LS_PBZ	16.67 ± 0.33^a^	0.61 ± 0.005^a^	0.35 ± 0.003^a^	3.65 ± 0.025^a^

*The different letters (as superscript) in a column denotes significant difference among the treatments within the given variety and stress level according to LSD test at P < 0.05. The value follows ± represent the standard error (n = 5).*

*TS, Timely sown; LS, Late Sown; C, Control; GA, GA3 spray; PBZ, Paclobutrazol spray*.

The mean value of stomatal conductance and transpiration rate in TS plants was 3.28 cm s^−1^ and 0.28 mmol cm^−2^ s^−1^, respectively. The imposition of stress conditions increased stomatal conductance (23% significant at *P* < 0.001) and transpiration rate (29% significant at *P* < 0.001). Among the cultivars, HD 2833 recorded the least stomatal conductance, and maximum was recorded by DBW 14 and HD 2643. GA_3_ had a non-significant (*P* < 0.05) effect on increased stomatal conductance except in HD 2643 under the stressed condition. There was a significant increase in stomatal conductance and transpiration rate on PBZ application under non-stressed conditions. However, stressed conditions could significantly (*P* < 0.05) affect stomatal conductance and transpiration rate in the cultivars HD2643 and HD2833.

### Membrane Stability Index and Lipid Peroxidation

**Figure 2:**
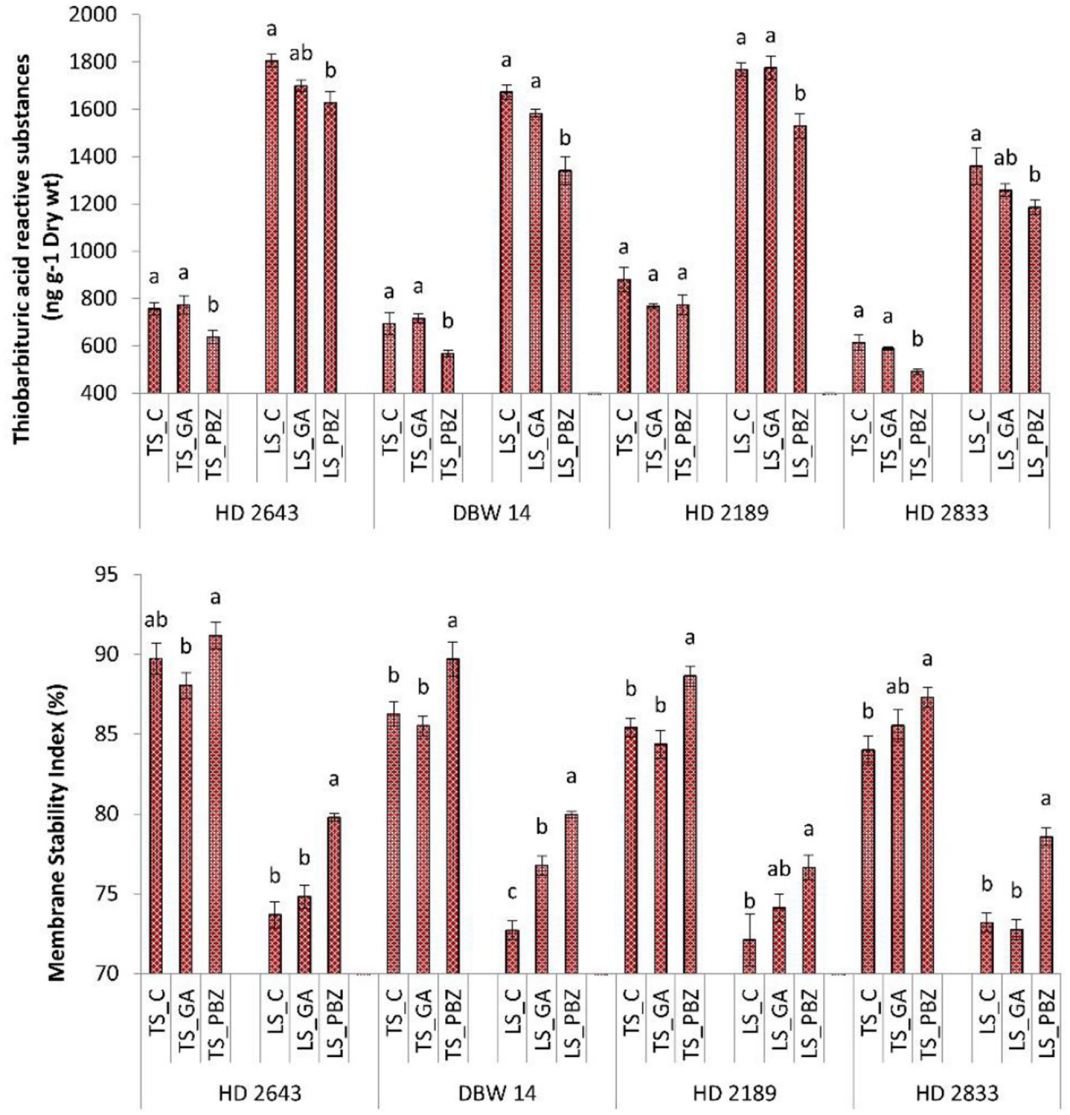
Effect of post-anthesis application of gibberellic acid (100 ppm) and paclobutrazol (50 ppm) on lipid peroxidation (TBARS content ng^−1^ dry wt); membrane stability index (%) in wheat exposed to timely and late sown conditions. The different letters on bar denotes significant difference among the treatments within the given variety and stress level according to LSD test at *P* < 0.05. The error bar represents the standard error. Value on the ordinate is the mean of five replicates. On the abscissa, HD 2643, DBW 14, HD2189, and HD2833 are varieties; TS and LS denote timely sown and late sown of stress treatment, respectively; C, GA, and PBZ denote control, GA3, and paclobutrazol of spray treatment, respectively.

The mean Membrane stability index (MSI) value varied from ~84 to 91% under control conditions, and it ranged from 72 to 79% under stress conditions (Figure 2). Heat stress as a main effect caused a significant (*P* < 0.001) reduction in MSI. The PBZ application resulted in a significant (*P* < 0.05) increase between 6 and 10% in MSI under a stressed environment. GA_3_ had a non-significant (*P* < 0.05) impact on the improvement of MSI under a non-stressed environment. However, it significantly (*P* < 0.05) increased MSI in cultivars DBW 14 under stress. The mean value of Lipid peroxidation (LPD) in plants under non-stressed conditions ranged from 500 to 770 nmol g^−1^ dry wt while it increased to 1,100–1,800 nmol g^−1^ dry wt under stressed conditions. It was about 100–150% higher than control plants under late sown conditions. In the PBZ application, the LPD content was significantly reduced between 10 and 24%. The effect of PBZ spray was most evident in DBW 14 (20%). GA_3_ application resulted in a non-significant (*P* < 0.05) reduction in LPD under the stressed condition.

### Antioxidant Enzymes Activity and H_2_O_2_ Content

The main effect of variety, stress, and spray showed significant (*P* < 0.001) impact on antioxidant enzymes system and H_2_O_2_ content (Supplementary Table 4). In LS plants of tolerant cultivars, mean value of SOD, APOX, CAT, and GR activity were increased by 121, 218, 201, and 62%, and an increase of 51, 121, 136, and 33% was recorded in susceptible cultivars, respectively (Table 3). Application of GA_3_ had a non-significant effect on SOD, APOX, CAT, and GR enzyme activity in LS plants of all the cultivars except SOD activity in HD 2643 at *P* < 0.05. However, PBZ significantly boosted the antioxidant defense mechanism in stressed plants. Under stress, PBZ increased mean value of SOD, APOX, CAT, and GR activity by 25.25, 16.8, 22.6, and 10.4% in tolerant cultivars, and 24, 22.6, 24.5, and 20.4% in susceptible cultivars, respectively at *P* < 0.05. The mean value of H_2_O_2_ content in plants under non-stressed condition was 0.9 μmol H_2_O_2_ g dry wt^−1^, while it was 1.54 μmol H_2_O_2_ g dry wt^−1^ under stressed conditions. H_2_O_2_ content was about 55–87% higher under heat stress as compared to non-stressed plants. PBZ reduced the H_2_O_2_ content significantly (*P* < 0.05) in all the cultivars under stressed conditions. The magnitude of reduction was 9 and 12% under non-stressed and stressed conditions, respectively. The effect of PBZ spray was most evident in cultivar DBW 14 and HD 2189 under stressed environmental conditions.

**Table 3:** Effect of post-anthesis application of gibberellic acid (100 ppm) and paclobutrazol (50 ppm) on superoxide dismutase content (unit min^−1^ mg^−1^ protein); glutathione reductase (Δ A_412_ min^−1^ mg^−1^ protein); ascorbate peroxidase (μmol ascorbate oxidized min^−1^ mg^−1^ protein); catalase activity (nmol H_2_O_2_ min^−1^ mg^−1^ protein); H_2_O_2_ content (μmol H_2_O_2_ g dry wt^−1^) in wheat exposed to timely and late sown conditions.

**Variety**	**Stress** **_Spray**	**Suseroxide dismutase** **(unit min** ^**–1**^ **mg** ^**–1**^ **protein)**	**Ascorbate peroxidase activity** **(μmol asc. oxidized** **min** ^**–1**^ **mg** ^**–1**^ **protein)**	**Catalase activity** **(nmol H**_**2**_O_**2**_**min**^**–1**^** mg**^**–1**^**protein)**	**Glutathione reductase** **(nmol H**_**2**_O_**2**_**min**^**–1**^ **mg**^**–1**^**protein)**	**H**_**2**_**O**_**2**_**content** **(μmol H**_**2**_O_**2**_**g** **drywt**^**–1**^**)**
HD 2643	TS_C	3.98 ± 0.21^a^	5.14 ± 0.14^b^	1.93 ± 0.03^b^	1.08 ± 0.03^b^	0.88 ± 0.03^a^
	TS_GA	4.23 ± 0.26^a^	6.9 ± 0.22^a^	1.91 ± 0.05^b^	1.09 ± 0.03^ab^	0.91 ± 0.03^a^
	TS_PBZ	4.65 ± 0.13^a^	6.99 ± 0.22^a^	2.16 ± 0.08^a^	1.21 ± 0.04^a^	0.94 ± 0.04^a^
	LS_C	9.13 ± 0.15^c^	13.01 ± 0.33^b^	6.4 ± 0.2^b^	1.67 ± 0.08^b^	1.49 ± 0.02^a^
	LS_GA	9.9 ± 0.15^b^	12.71 ± 0.69^b^	6.37 ± 0.12^b^	1.76 ± 0.06^b^	1.44 ± 0.05^a^
	LS_PBZ	9.61 ± 0.41^a^	15.13 ± 0.66^a^	7.5 ± 0.08^a^	1.89 ± 0.02^a^	1.32 ± 0.04^b^
DBW 14	TS_C	5.2 ± 0.07^c^	3.41 ± 0.4^b^	2.05 ± 0.04^a^	1.02 ± 0.06^a^	0.88 ± 0.05^a^
	TS_GA	5.75 ± 0.13^b^	3.16 ± 0.31^b^	2.1 ± 0.06^a^	0.99 ± 0.06^a^	0.8 ± 0.05^ab^
	TS_PBZ	6.65 ± 0.23^a^	5.59 ± 0.31^a^	2.08 ± 0.1^a^	1.08 ± 0.05^a^	0.71 ± 0.03^b^
	LS_C	11.15 ± 0.25^b^	13.07 ± 0.41^b^	5.54 ± 0.07^b^	1.72 ± 0.01^a^	1.36 ± 0.01^a^
	LS_GA	10.43 ± 0.41^b^	13.88 ± 0.64^ab^	4.72 ± 0.33^b^	1.67 ± 0.03^a^	1.25 ± 0.05^b^
	LS_PBZ	14.57 ± 0.63^a^	15.35 ± 0.42^a^	7.11 ± 0.5^a^	1.85 ± 0.1^a^	1.18 ± 0.03^b^
HD 2189	TS_C	4.33 ± 0.32^b^	6.4 ± 0.27^b^	2.09 ± 0.03^a^	1.03 ± 0.05^a^	0.85 ± 0.02^a^
	TS_GA	4.6 ± 0.4^b^	6.99 ± 0.32^ab^	2.06 ± 0.05^a^	1.08 ± 0.01^a^	0.79 ± 0.02^ab^
	TS_PBZ	5.87 ± 0.15^a^	7.73 ± 0.27^a^	2.11 ± 0.14^a^	1.08 ± 0.05^a^	0.75 ± 0.04^b^
	LS_C	5.76 ± 0.32^b^	11.19 ± 0.64^b^	4.66 ± 0.21^b^	1.41 ± 0.03^b^	1.6 ± 0.08^b^
	LS_GA	6.12 ± 0.15^b^	11.13 ± 0.23^b^	4.97 ± 0.14^b^	1.47 ± 0.02^b^	1.81 ± 0.06^a^
	LS_PBZ	7.49 ± 0.18^a^	14.33 ± 0.32^a^	6.12 ± 0.21^a^	1.62 ± 0.04^a^	1.39 ± 0.01^c^
HD 2833	TS_C	4.2 ± 0.21^b^	2.84 ± 0.19^b^	2.07 ± 0.05^a^	1.03 ± 0.06^a^	1 ± 0.1^a^
	TS_GA	4.87 ± 0.15^b^	2.68 ± 0.27^b^	2.26 ± 0.04^a^	1.03 ± 0.06^a^	0.93 ± 0.03^a^
	TS_PBZ	6.69 ± 0.31^a^	3.64 ± 0.13^a^	2.32 ± 0.14^a^	1.05 ± 0.04^a^	0.81 ± 0.04^a^
	LS_C	7.12 ± 0.16^b^	7.65 ± 0.35^b^	5.15 ± 0.04^b^	1.35 ± 0.03^b^	1.69 ± 0.03^a^
	LS_GA	7.6 ± 0.36^ab^	7.91 ± 0.42^ab^	5 ± 0.16^b^	1.37 ± 0.03^b^	1.78 ± 0.06^a^
	LS_PBZ	8.4 ± 0.15^a^	8.98 ± 0.18^a^	6.07 ± 0.29^a^	1.7 ± 0.08^a^	1.5 ± 0.07^b^

*The different letters (as superscript) in a column denotes significant difference among the treatments within the given variety and stress level according to LSD test at P < 0.05. The value follows ± represent the standard error (n = 5).*

*TS, Timely sown; LS, Late Sown; C, Control; GA, GA3 spray; PBZ, Paclobutrazol spray*.

### Relative Gene Expression

**Figure 3:**
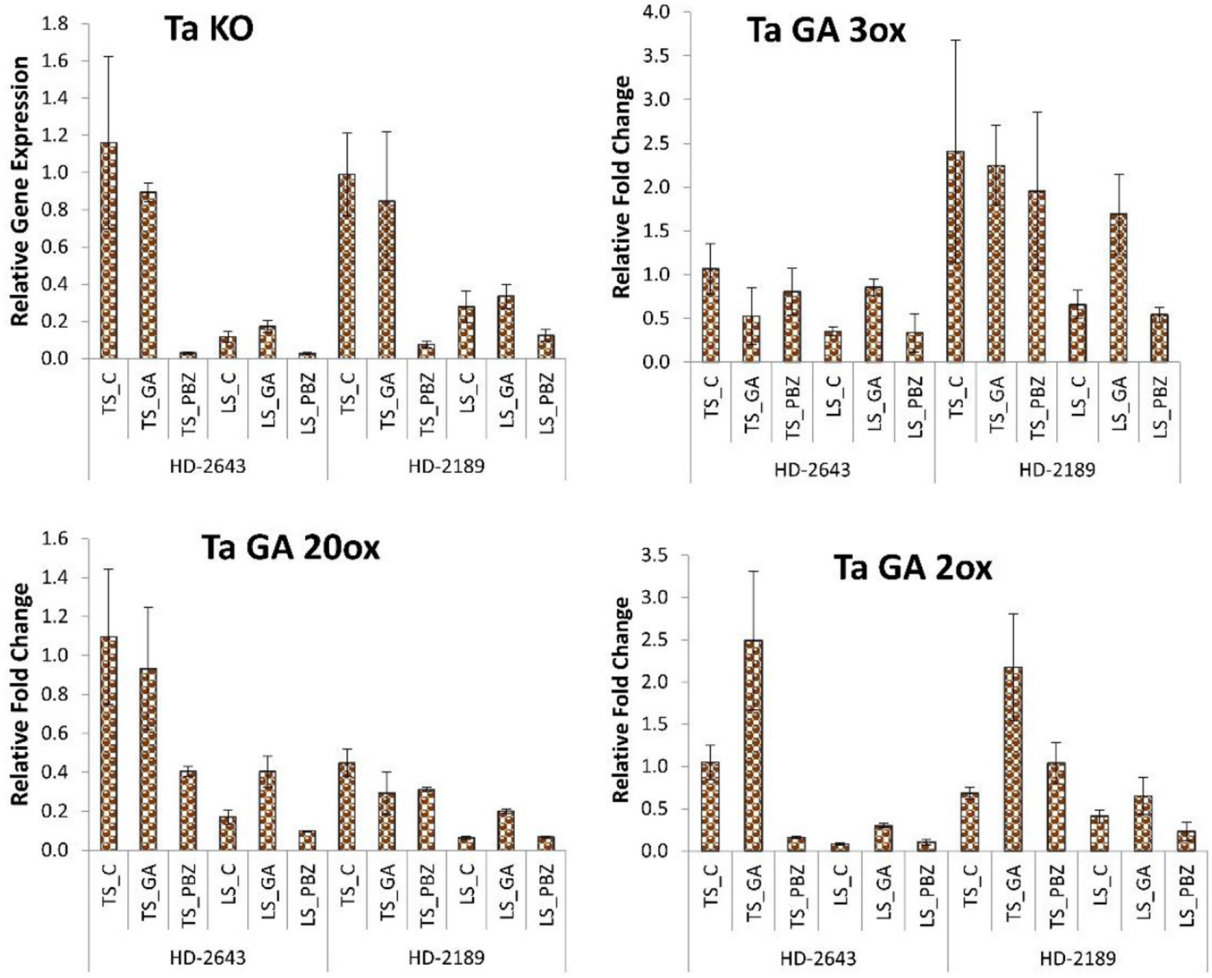
Relative fold change in expression of GA biosynthesis and degradation genes, i.e., Kaurene oxidase (KO), GA 3oxidase, GA 20 oxidase, and GA 2 oxidase in developing grain. The error bar represents the standard error. Value on the ordinate is the mean of three replicates. On the abscissa, HD 2643 and HD 2189 are varieties; TS and LS denote timely sown and late sown of stress treatment, respectively; C, GA, and PBZ denote control, GA3, and paclobutrazol of spray treatment, respectively.

In the RT-PCR study, the genes of GA biosynthesis and degradation pathways showed a significant change in gene expression level under different stress environments on the application of GA_3_ and PBZ. The genes that showed a significant change in their RT-PCR expression study were selected for the RT-qPCR study (Supplementary Figure 1). Application of GA_3_ slightly decreased KO expression, GA20ox, and GA3ox in non-stressed plants, whereas in stressed plants, upregulation in gene expression level was observed (Figure 3). The expression level of GA degrading enzyme GA2ox was upregulated on GA_3_ application in both cultivars, but the increase in expression was very high in TS plants compared to LS plants. Expression of GA degrading enzyme GA2ox was upregulated in both cultivars understudied environmental conditions on GA_3_ application, but the increase was very high in TS plants compared to LS. PBZ application drastically decreased the mRNA level of KO by 10–20 times. The PBZ application also significantly decreased gene expression of other genes like GA20ox, GA3ox, and GA 2ox.

**Figure 4:**
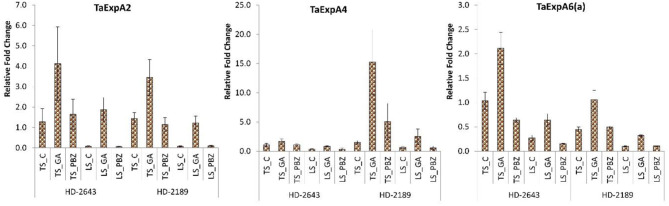
Relative fold change in expression of wheat cell expansin genes, i.e., Ta Exp 2, Ta Exp A4, and Ta Exp A6(a) in developing grain. The error bar represents the standard error. Value on the ordinate is the mean of three replicates. On the abscissa, HD 2643, and HD 2189 are varieties; TS and LS denote timely sown and late sown of stress treatment, respectively; C, GA, and PBZ denote control, GA3, and paclobutrazol of spray treatment, respectively.

The relative gene expression level of Ta EXp A2 and Ta Exp A6(a) was higher in tolerant cultivar HD 2643, and Ta Exp A4 was higher in the susceptible cultivar. Under stress conditions, the expression of all three genes was downregulated. The Ta Exp A2 gene expression had a maximum decrease under stress. Expression of all three studied cell expansins gene, i.e., Ta Exp 2, Ta Exp A4, and Ta Exp A6(a) was upregulated on GA_3_ application under both conditions. Expression of Ta ExpA2 showed a maximum increase, and an increase in expression was higher under stressed condition on application of GA_3_ (Figure 4).

## Discussion

Abiotic stress leads to alteration in phytohormones levels and decreased plant growth (Abdulaziz et al., 2020). Stress conditions alter hormonal homeostasis, stability, content, biosynthesis, and compartmentalization in the plant. Abiotic stresses like salinity, cold, and drought also cause a decrease in endogenous GA level (Yang et al., 2001; Achard, 2006; Magome et al., 2008; Tuna et al., 2008; Alonso-Ramírez et al., 2009; Ahmad, 2010). Akin to other stresses, our study also observed a reduction in endogenous GA_3_ content under terminal heat stress in studied wheat cultivars. We report a decrease in transcript level of GA biosynthesis pathway genes, i.e., CPS, EKS, KO, KAO, GA20ox, GA3ox, and GA2ox under stress.

The expression study results supported our finding that reduced GA content was due to decreased biosynthesis of gibberellin, even though the transcript level of GA degrading enzyme GA2ox decreased under stress. The decrease in GA2ox activity could have prevented the further degradation of bioactive gibberellins so that plants could maintain a minimum level of endogenous gibberellins under stress.

Exogenous gibberellins/PBZ may regulate the level of active gibberellins in the plant (Tonkinson et al., 1997). We observed that exogenous spray of GA_3_ and PBZ significantly (*P* < 0.05) induced and inhibited endogenous GA_3_ level, respectively, in tolerant and susceptible cultivars. Susceptible and tolerant varieties did not respond differently toward the application of both growth regulators. PBZ inhibited KO, a cytochrome P450 monooxygenase class enzyme of the GA biosynthesis pathway (Tuna et al., 2008); thus, a highly significant decrease in the KO enzyme transcript level was seen in all cultivars on PBZ application. Inhibition of this enzyme significantly also reduced the level of endogenous GA in plants. Baninasab and Ghobadi (2011) reported downregulation of KO, GA20ox, and GA3ox under ambient and high-temperature conditions on PBZ application. A relatively more significant decrease in GA biosynthesis was observed in TS plants due to PBZ application. The higher decrease in expression level of GA biosynthesis genes is may be attributed to the more active GA biosynthesis mechanism of TS plants than in LS plants. GA_3_ application upregulated GA2ox (GA degrading enzyme) expression in both types of cultivars, and it can be inferred that over-accumulation of bioactive gibberellin induced negative feedback regulation and increased its degradation to maintain the optimum level of hormone. In contrast, PBZ application downregulated the expression of GA2ox further to reduce the degradation of 656 bioactive gibberellins in plants. Tuna et al. (2008) also observed a decrease in expression of GA2ox activity on PBZ application under salinity stress.

Generally, under stress conditions, simultaneous ROS generation and consequent enhanced antioxidant system activity have been observed in plants (Almeselmani et al., 2009; Wang et al., 2014). The trade-off between ROS generation and the antioxidant enzyme system decides the tolerance ability of the plant under stress. In conformity to previous studies, we also observed an increase in ROS, i.e., under the heat-stressed environment in all cultivars, H_2_O_2_ had a relatively higher increase in susceptible cultivars than tolerant cultivars. In tolerant cultivars, activity of antioxidant enzymes like superoxide dismutase, ascorbate peroxidase, peroxidase, catalase, and glutathione reductase was recorded higher in comparison to susceptible cultivars. The increase in ROS such as superoxide, hydroxyl ion, and hydrogen peroxide may be attributed to an increase in free-electrons for O_2_ due to excessive excitation of photosystems. The increased ROS level has various effects on peroxidation of membrane lipid and accumulation of malondialdehyde compound, such as increases in electrolyte leakage from the cell, leading to damage to the membrane (Senaratna et al., 1988; Hasanuzzaman et al., 2020) and PBZ-induced stress protection through an efficient antioxidant mechanism (Zhou and Leul, 1998; Soumya et al., 2017). To date, comparative analysis of exogenous PBZ/GA_3_-mediated stress tolerance through GA_3_ biosynthesis inhibition/induction has been less explored. We observed that PBZ conferred stress protection by reducing oxidative damage caused by ROS through an increased level of antioxidant enzymes, but not through GA_3_ biosynthesis inhibition. The observation further supported that exogenous GA_3_-mediated GA_3_ biosynthesis induction did not significantly affect the activity of the antioxidant enzymes.

Erstwhile studies have shown a significant decrease in electrolyte leakage of susceptible wheat varieties as compared to tolerant varieties (Almeselmani et al., 2009). The electrical conductivity of the leachate from heat-shocked seedlings of susceptible cultivars was higher than seedlings of tolerant cultivars grown in a controlled environment (ElBasyoni et al., 2017). Similarly, we also observed higher Malonaldehyde (MDA) accumulation and an increase in electrolyte leakage of LS plants. The PBZ application increased membrane thermostability by reducing lipid peroxidation-mediated damage to the unsaturated lipid of the membrane. Such plants could scavenge more ROS generated under stress due to high availability of the antioxidant enzymes (Chakraborty and Tongden, 2005). In general, no significant effect of GA_3_ application was seen on lipid peroxidation and electrolyte leakage. It could be due to the non-significant effect of GA_3_ on antioxidant enzyme activity. On the contrary, electrolyte leakage was enhanced by GA_3_ foliar application under salinity by Tuna et al. (2008). Our results indicate that a higher level of endogenous GA_3_ is not associated with an antioxidant defense system and membrane stability.

High-temperature damages to the photosystem electron transport chain reduced the activity of rubisco and rubisco activase. The solubility of CO2 decreases under high temperature. It increases photorespiration, thus reducing photosynthesis rate under stress (Wahid et al., 2007; Parry et al., 2011). Photochemical efficiency is the ratio of variable to maximum fluorescence (Fv/Fm), an indicator of photosystem II efficiency, which positively correlates with photosynthesis rate. Thus, a higher Fv/Fm ratio is associated with enhanced stress tolerance (Parry et al., 2011; Sharma et al., 2015; Faseela et al., 2020). We found that photosynthesis rate and Fv/Fm ratio decreased in plants under LS condition. The decrease in photosynthesis rate and Fv/Fm ratio was found to be cultivar-dependent. Tolerant cultivars maintained a higher photosynthesis rate and Fv/Fm ratio in a stressed environment than susceptible ones. In tolerant cultivars, the presence of more efficient photosystems and heat stress-tolerant enzymes of photosynthesis, i.e., rubisco and rubisco activase, have been reported previously (Feng et al., 2014; Brestic et al., 2018). In PBZ-treated plants, photosynthesis rate and Fv/Fm ratio were higher than those of non-treated plants under LS conditions. These results were in agreement with the findings of previous researchers (Mahoney et al., 1998; Sharma et al., 2015). Results indicated that PBZ could decrease the heat-induced photo-inhibition by protecting photosystem II. The PBZ application increased photosynthesis rate by reducing ROS-mediated damage to photosynthesis machinery and photosynthetic pigments in plants. Transpiration rate and stomatal conductance were higher in stressed plants as compared to their respective control. Our results were also in agreement with the findings of Feng et al. (2014) and Sharma et al. (2015). The GA_3_/PBZ application did not affect stomatal conductance and transpiration rate under both temperature conditions in studied cultivars. Thus, we concluded that the decline in photosynthesis rate under heat stress was not the consequence of stomatal limitation.

LS plants were exposed to different temperatures and photoperiods during their life cycle compared to TS plants, which was a deviation from standard requirement (Nagar et al., 2015). This reduced plant growth and development, resulting in diminished height, tiller number, and dry matter accumulation. High temperature increased the rate of development, but at the same time, reduced the duration of crop growth and grain filling. The compensatory effect of grain filling rate was not adequate to overcome the decrease in duration, leading to a decrease in seed setting and 1,000 grain weight, ultimately causing a significant reduction in yield (Reynolds et al., 2012; Tan et al., 2015). High temperature diverted the photo-assimilates from developing stress tolerance mechanism to survive under stress, which led to a decrease in the availability of photo-assimilates for the development of reproductive organs (Wahid et al., 2007; Janda et al., 2019; Hasanuzzaman et al., 2020). The rate of starch deposition in wheat grains was decreased by >30% at temperatures between 30 and 40°C, with the early grain filling being the most critical stage (Stone and Nicolas, 1994). Reduction in growth parameters and yield traits was higher in susceptible cultivars than tolerant cultivars because of stress condition. Decreases in active photosynthesis area and the photosynthesis rate were less in tolerant cultivars than sensitive cultivars. Application of PBZ and GA_3_ had no significant effect on growth parameters. At anthesis, growth regulators applied, but by that time, the plant had already achieved its maximum growth. In contradiction, Khan and Samiullah (2003) also observed an increase in total dry matter in *Brassica juncea* when 10^−5^M GA3 was applied at 40 days after sowing.

The smaller grain size under stress reduces grain weight potential, leading to a significant decrease in final grain yield. Grain weight depends on the number and size of cells in the pericarp and endosperm; it determines the sink capacity of the grain (Kaur et al., 2011). Additionally, cell size plays a more significant role than cell numbers in developing endosperm and pericarp, as they determine the grain expansion ability during grain development. Cell expansion enzymes also play a determining role in the process of cell wall extension and cell size (McQueen-Mason et al., 1992; Lizana et al., 2010). We reported a decrease in mRNA level of expansin proteins, i.e., TaExp A2, TaExp A4, and TaExpA6a under stress. Thus, this decrease reduced the cell expansin enzyme activity; which, in turn, decreased cell size in pericarp and endosperms, limited the accumulation of starch in endosperm, and reduced the grain size. Smaller grains and a decrease in test weight under stress might also be due to decreased cell expansins enzyme activity. The increase in endogenous gibberellic acid level in developing grains might have contributed to a gain in 100 seed weight, as the spray of GA_3_ coincided with the most sensitive period of grain weight determination. Gibberellins regulate grain filling duration in crop plants, contributing to individual grain weight (Wang et al., 2006), and GA_3_ is also associated with the grain filling process, like an increase in mobilization of storage reserve, which leads to an increase in mean grain weight. Furthermore, Yang et al. (2001) revealed a positive correlation between content of the gibberellins and the rate of embryo development during the grain filling stage in rice. We reported the increase in transcript level of three cell expansin genes, i.e., TaExp A2, TaExp A4, and TaExp A6(a), in developing seed on exogenous application of GA_3_. Choi et al. (2006) reported an increase in transcript level of cell wall expansins gene OsEXP4 after 30 min of exogenous application of gibberellin in rice. Our results also demonstrated that GAs contents in grains were positively related to the increased grain weight, which caused an increase in grain storage space due to an increase in pericarp cell expansion rate and endosperm cell division. This was caused by an increase in cell expansins gene expression.

In general, PBZ application improved the grain yield and 1,000-seed weight with a relatively more significant increase in susceptible cultivars. The efficient antioxidant defense system of PBZ-treated plants contributed to a higher photosynthesis rate under stress and, consequently, increased photo-assimilate availability for grain development. PBZ did not affect the cell expansin genes activity, which implied that the cell expansins-mediated mechanism does not play a role in PBZ-treated plants. Harvest index showed the non-significant effect on GA_3_/PBZ application, which suggests that GA_3_ and PBZ were unable to change the ratio of grain yield to biomass, agreeing with the results of Baninasab and Ghobadi (2011). The study concludes that the inhibitory/inducing effect of PBZ/GA_3_ application on endogenous GA_3_ level is not solely responsible for the thermo-protection under terminal heat stress in wheat. We report that an increased level of endogenous GA_3_ did not significantly influence the antioxidant enzyme activity thus indicates that PBZ would have provided thermotolerance through another mechanism instead of the GA_3_ inhibitory mechanism. Other mechanisms of conferring stress tolerance by PBZ could be through the enhanced synthesis of proline, abscisic acid, and salicylic acid biosynthesis (Nivedithadevi et al., 2012; Khan et al., 2015; Soumya et al., 2017; Kousar et al., 2018). We reported that an increase in levels of endogenous GA_3_ brought no change in thermotolerance traits, but had a positive effect on grain yield and test weight. An increase in cell expansin genes activity might have, in part, contributed to an increase in individual grain weight.

## Data Availability Statement

The original contributions presented in the study are included in the article/Supplementary Material, further inquiries can be directed to the corresponding author/s.

## Author Contributions

VS, AA, and GS proposed the hypothesis and designed the experiment. SN and RSR conducted the experiment. SN, SK, and RD analyzed the data and wrote the manuscript. SN and NS standardized the GA extraction protocol. All authors contributed to the article and approved the submitted version.

## Conflict of Interest

The authors declare that the research was conducted in the absence of any commercial or financial relationships that could be construed as a potential conflict of interest.

## Publisher's Note

All claims expressed in this article are solely those of the authors and do not necessarily represent those of their affiliated organizations, or those of the publisher, the editors and the reviewers. Any product that may be evaluated in this article, or claim that may be made by its manufacturer, is not guaranteed or endorsed by the publisher.
