# Supplementary material for: Understanding the Role of Gibberellic Acid and Paclobutrazol in Terminal Heat Stress Tolerance in Wheat
Source: Front Plant Sci. 2021 Aug 19;12:692252. doi: 10.3389/fpls.2021.692252 (PMC8417122; doi:10.3389/fpls.2021.692252)
Supplement: Supplementary file 1 [file Data_Sheet_1.PDF]

Understanding the role of gibberellic acid and paclobutrazol in terminal heat stress tolerance in wheat

Supplementary Material

Table S1: Temprature (°C) for wheat growing season of year 2014-15

SMW	T Max (°C)	T Min (°C)	T Avg (°C)
2014			
46	27.0	8.0	17.5
47	27.0	7.1	17.1
48	27.9	10.4	19.2
49	27.0	9.0	18.0
50	21.3	7.7	14.5
51	16.0	5.7	10.9
52	16.6	3.6	10.1
2015			
1	17.9	8.7	13.3
2	15.1	5.2	10.2
3	18.5	5.3	11.9
4	16.1	9.1	12.6
5	20.0	7.6	13.8
6	22.6	6.7	14.7
7	24.7	9.9	17.3
8	27.4	14.8	21.1
9	23.7	11.7	17.7
10	24.1	9.6	16.9
11	25.5	12.9	19.2
12	29.7	13.5	21.7
13	32.9	17.8	25.4
14	30.2	17.0	23.6
15	31.9	17.7	24.8
16	36.5	20.5	28.5
17	37.2	21.3	29.3
18	38.5	20.9	29.8

*SMW- standard meteorological week

Table S1: Average Temprature (°C) faced during vegetative and reproductive stage by wheat.

		Tmax(°C)	Tmin (°C)	Tavg (°C)
**Vegetative phase**				
Average temperature	Timely sown	21	7.24	14.13
	Late sown	20.28	8.41	14.36

	Rise in temp	0.72	-1.17	-0.23
**Reproductive phase**				
Avg. temperature of 10 days at 50% anthesis	Timely sown	27.0	14.6	20.9
	Late sown	32.7	16.6	24.7
	Rise in temp	5.71	1.95	3.82
Avg. temperature of grain filling duration from the date of 50% anthesis	Timely sown	26.51	12.68	19.62
	Late sown	32.87	18.25	25.58
	Rise in temp	6.36	5.57	5.96

Table S3: Details of primer sequence and NCBI gene accession no.

Gene	Primer sequence	Primer (bp)	NCBI gene accession no
TaExpA2	F>>CCACCATGATGTGTTGTTCC<br>R>>AGTAGGAGTGGCCGTTGATG	20<br>21	FN556065.1
TaExpA4	F>>AACTTCTGCCCGTCGAACTA<br>R>>CCCTTCATGGTGAACCTCAT	20<br>20	FN556066.1
TaExpA6(a)	F>>GTGCAACCCTCCTCGACAC<br>R>>GGTCCCCTTCACCGACAT	19<br>18	FN556070.1
TaEks	F>>CCAACAAAGTACAACGATGTGAGC<br>R>>CGTGATGTTCGAAAGCACCTG	24<br>21	AB597958.1
TaCPS D	F>>TGTTGGTGGAGGACCAGTGTAC<br>R>>GTCTCGTTCGTTTTGGTGTCCTG	22<br>23	GU980888.1
TaKO	F>>CAAGGTTCGTCCATGAGAACAC<br>R>>TAGCTGGTGAGTTGCACAGTGTC	22<br>23	GU980895.1
Ta KAO	F>> CGGTTCGTCAACATCTCCTTC<br>R>>TGTCAGCTTGTAGCCAAGGAG	21<br>21	GU980892.1
$TaGa_2Ox$	F>>CGCGCATACGTACCATATT<br>R>>TTGACGAGCTTGAAGAACC	19<br>19	LN828682.1
$TaGa_3Ox$	F>>GATATCGCTCGGCTACTTCCTC<br>R>>CGATCTACGACGAGATCAGGTC	22<br>22	LN828690.1
$TaGa_{20}Ox$	F>>GACATGATGGATTGCTCTGCTC<br>R>>ACGTGCTACCACTAGATCAACC	22<br>22	FR716527.1

Table S4: The P-value obtained from three factorial Analysis of variance (ANOVA) for completely randomized design (CRD) consisting genotype, stress and spray as fixed factors for all the physiological, biochemical, growth and yield traits.

	Variety	Spray	Stress	Variety * Spray	Variety * Stress	Spray * Stress	Variety * Spray * Stress
Grain yield	0.0000	0.0000	0.0000	0.7578	0.0000	0.0581	0.0167

Membrane Stability Index	0.0000	0.0000	0.0000	0.6972	0.0093	0.0015	0.0448
Lipid Peroxidation	0.0000	0.0000	0.0000	0.4634	0.0000	0.0161	0.0477
Superoxide Dismutase	0.0000	0.0000	0.0000	0.0021	0.0000	0.0265	0.0000
Ascorbate peroxidase activity	0.0000	0.0000	0.0000	0.2236	0.0000	0.0392	0.0138
Catalase activity	0.0000	0.0000	0.0000	0.1716	0.0000	0.0000	0.0272
H_2O_2 content	0.0000	0.0000	0.0000	0.0035	0.0000	0.0015	0.0206
Glutathione reductase	0.0000	0.0000	0.0000	0.6395	0.0000	0.0034	0.3608
Peroxidase	0.0000	0.0000	0.0000	0.0008	0.0000	0.0000	0.0172
Plant height	0.0000	0.4808	0.0000	0.9986	0.9974	0.7706	0.9609
Test weight	0.0000	0.0000	0.0000	0.0000	0.0000	0.0000	0.0000
Ear/Plant	0.4587	0.6518	0.0000	0.9921	0.8448	0.6234	0.9361
Grain number/ ear	0.0000	0.0000	0.0000	0.1971	0.0022	0.0035	0.0415
Grain weight/ ear	0.0000	0.0000	0.0000	0.2964	0.0000	0.0565	0.0090
Harvest Index	0.0000	0.0381	0.0000	0.8657	0.0003	0.9131	0.6905
Total dry matter	0.0000	0.0208	0.0000	0.8568	0.1552	0.1890	0.8033
Tiller Number	0.0065	0.6525	0.0023	0.8949	0.2272	0.7828	0.7956
Photosynthesis rate	0.0000	0.0000	0.0000	0.5677	0.7823	0.4651	0.0085
Fv/Fm ratio	0.0000	0.0000	0.0000	0.0000	0.0000	0.4924	0.0002
Transpiration rate	0.0000	0.0000	0.0000	0.9652	0.0000	0.1847	0.0347
Stomatal Conductance	0.0000	0.0000	0.0000	0.0008	0.0000	0.0324	0.0095
Gibberelic Acid (GA3)	0.0000	0.0000	0.0000	0.0001	0.0000	0.0001	0.0928

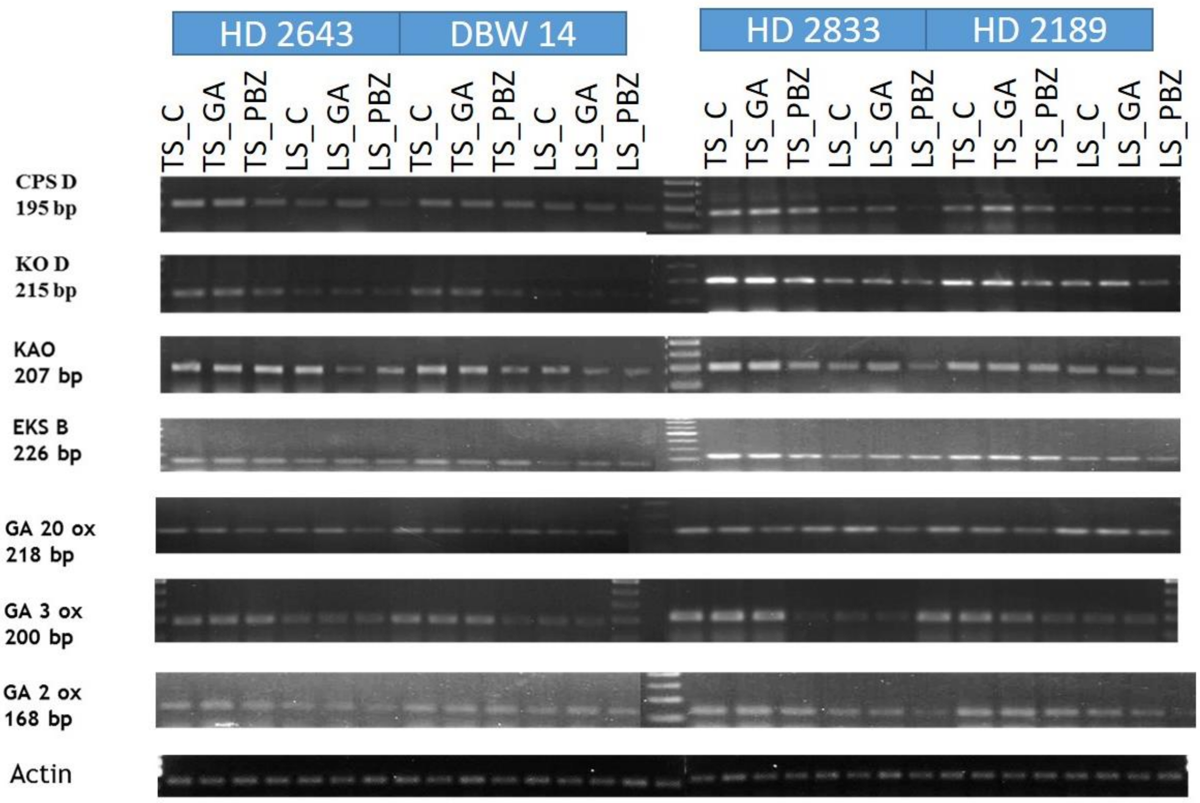

Figure S 1. RT-PCR gene expression of GA biosynthesis and degradation pathway genes.
